# Precise Robotic Y-V Plasty for Vesico-Urethral Anastomotic Stenosis: Avoiding Urinary Incontinence

**DOI:** 10.1590/S1677-5538.IBJU.2026.0221

**Published:** 2026-08-05

**Authors:** Lin Wang, Fuhao Ji, Chao Yang, Chen Zhu, Yinglong Sa, Yidong Liu, Xiangguo Lv

**Affiliations:** 1 Shanghai Jiao Tong University School of Medicine Renji Hospital Shanghai China Department of Urology, Renji Hospital, School of Medicine, Shanghai Jiao Tong University, Shanghai, China; 2 The Second Hospital of Anhui Medical University Department of Urology Hefei China Department of Urology, The Second Hospital of Anhui Medical University, Hefei, China; 3 Jiangsu Province Hospital of Chinese Medicine Department of Urology Nanjing China Department of Urology, Jiangsu Province Hospital of Chinese Medicine, Nanjing, China, Nanjing, China; 4 Shanghai Jiaotong University Affiliated Sixth People's Hospital Department of Urology Shanghai China Department of Urology, Shanghai Jiaotong University Affiliated Sixth People's Hospital, Shanghai, China

**Keywords:** Prostatectomy, Robotic Surgical Procedures, Urinary Incontinence

## Abstract

**Objective:**

The treatment of vesico-urethral anastomotic stenosis (VUAS) is difficult because of the high risk of urinary incontinence. Cystoscopy-guided robotic Y-V plasty is hypothesized to reduce the incidence of urinary incontinence compared to traditional Y-V plasty. The purpose of this study was to evaluate the efficacy of cystoscopy-guided robotic Y-V plasty.

**Materials and Methods:**

All patients diagnosed with VUAS following radical prostatectomy between January 2019 and December 2024 were retrospectively reviewed. Patients with damaged external urethral sphincter or stenosis involving the external sphincter were excluded. Cystoscopy-guided robotic Y-V plasty was performed at least three months after the last failed intervention. After catheter removal, patients were scheduled for re-examination at one, six, twelve and twenty-four months. Uroflowmetry was routinely performed and cystoscopy was performed at six months after surgery. Postoperative changes in patients’ erectile function, lower urinary tract symptoms, and urinary continence were followed up using Five-item International Index of Erectile Function (IIEF-5), International Prostate Symptom Score (IPSS), and urinary continence grading.

**Results:**

Twenty-eight patients were enrolled. All patients were followed up for 6–59 (median 30) months after surgery. The final success rate was 96.4% (27/28). Normal continence was achieved in 25 patients (92.6%). Social continence occurred in 2 (7.4%). No patients experienced clinical incontinence. The patients’ postoperative IPSS scores achieved significant improvement compared with preoperative.

**Conclusion:**

The surgical strategy cystoscopy-guided robotic Y-V plasty is more precise, direct and efficient in protecting the external urethral sphincter. It ensures a high success rate and simultaneously reduces the occurrence of urinary incontinence.

## INTRODUCTION

Vesico-urethral anastomotic stenosis (VUAS) is a potential complication after radical prostatectomy (1). The reported incidences have significantly decreased since the advent of robotic surgery, largely thanks to higher-quality vesico-urethral anastomosis and greater water tightness (2-4). The actual incidence of VUAS has declined significantly over time, from an initial alarming rate of > 17% to a contemporary lower rate of < 1% (5, 6). Despite its decreasing prevalence, VUAS continues to pose a significant challenge in terms of management for both patients and reconstructive surgeons.

In case of stenosis recurrence after two or more failed endoscopic attempts, it is described as recalcitrant VUAS. This rare yet challenging condition lacks a universally accepted standard treatment approach. The first-line treatment often involves endoscopic procedures (7), with variations in its modalities: dilatation, incision, resection, or application of scar modulators. Surgical reconstruction is typically utilized for intricate cases of recurrent VUAS (8). Several reports advocate a vesico-urethral reanastomosis after failure of endoscopic treatments (9, 10). Nonetheless, the occurrence of new onset incontinence is surprisingly high, reaching up to 31%. This limits its widespread application. In 1950s, Young first described an Y-V plasty of the bladder neck (11). The Y-V plasty offers a high success rate by enabling reconstruction of a wide reanastomosis with a flap of healthy tissue. Although this technology brings new hope for bladder neck contracture after transurethral surgery for benign prostatic hyperplasia (12, 13), it is difficult to effectively treat the VUAS after radical prostatectomy. The primary challenge lies in mitigating the risk of urinary incontinence, given the limited length of the residual functional urethra following radical prostatectomy, which poses a challenge in avoiding damage to the external urethral sphincter. Therefore, avoiding intraoperative injury to the external sphincter is crucial to reduce the occurrence of new onset incontinence.

Robotic bladder neck Y-V plasty under direct vision of the cystoscope can achieve the goal of preserving urinary control function. Here, through this multi-center retrospective study, we aimed to share our experience and preliminary outcomes of cystoscopy-guided robotic Y-V plasty for the treatment of VUAS following radical prostatectomy.

## MATERIALS AND METHODS

### Patients and preoperative preparations

This study adheres to the principles outlined in the Declaration of Helsinki and was approved by the institutional review board (LY2025-378-B). Informed consent was obtained from all patients. All patients diagnosed with VUAS following radical prostatectomy between January 2019 and December 2024 were retrospectively reviewed. The surgeries were performed by four surgeons in three institutions. Preoperative evaluation included a medical history, physical examination, urinalysis, urine culture, uroflowmetry, retrograde and voiding cysto-urethrography (RVCUG), cystoscopy and magnetic resonance imaging (MRI). Based on the preoperative MRI (Figure-1A), RVCUG (Figure-1B) and cystoscopy (Figure-1C), patients who had undergone cystoscopy-guided robotic Y-V plasty and whose external urethral sphincter was structurally and functionally intact were included. However, those with damaged external urethral sphincter or stenosis involving the external sphincter were excluded. Patients who underwent radiotherapy after radical prostatectomy were excluded. In addition, patients with incomplete clinical records, missing follow-up data, or lost to follow-up were also excluded. Before surgery, the erectile functions of all patients were evaluated using the Five-item International Index of Erectile Function (IIEF-5). According to the IIEF-5, erectile function can be divided into normal, mild, moderate, and severe erectile dysfunction (ED) (14). The International Prostate Symptom Score (IPSS) was used to assess patients’ lower urinary tract symptoms (15). Cystoscopy-guided robotic Y-V plasty was performed at least three months after the last failed intervention.

**Figure 1:**
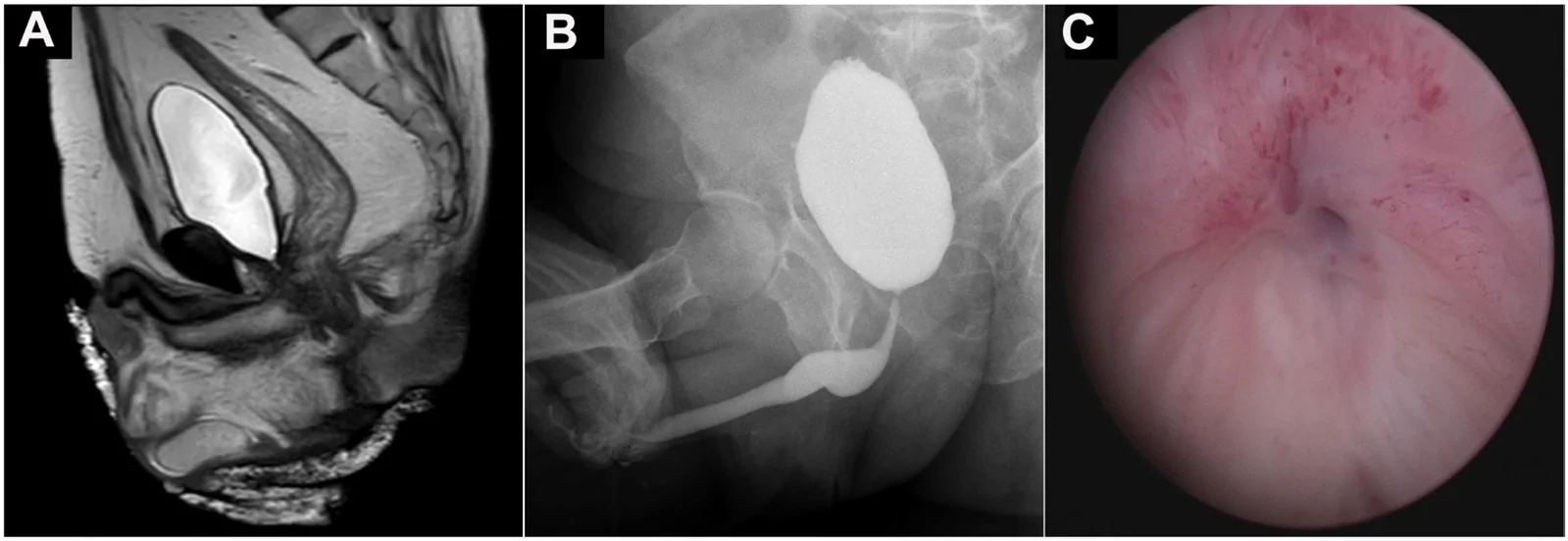
Preoperative evaluation by MRI.

A) RVCUG, B) and cystoscopy, C) of one patient. The distance between the bladder neck and the external sphincter is assessed by MRI; the length and degree of stenosis are assessed by RVCUG; and the distance between the bladder neck and the external sphincter and the degree of stenosis are further confirmed by cystoscopy.

## SURGICAL STRATEGY AND TECHNIQUES

The robotic surgery was performed using da Vinci Xi platform. Patients were placed in a low lithotomy position. The transabdominal robotic technique with five trocars began with entering the abdomen with an approach similar to a radical prostatectomy (16). The surgical procedure begins with the dissection and mobilization of the Retzius space. The bladder is first freed from the overlying peritoneum to identify the obstructed bladder neck under the guidance of cystoscopy performed by an auxiliar surgeon (Figures 2A and E). A cystoscope is placed at the membranous urethra and crosses the external urethral sphincter to confirm the integrity of external urethral sphincter and observe the incision of narrowed segment performed using monopolar scissors (Figures 2B and F). Then a Y-shaped incision of anterior bladder neck is performed (Figure 2C) under the observation of cystoscopy to avoid any potential injury to the external sphincter (Figure-2G and Supplementary video 1).

**Figure 2:**
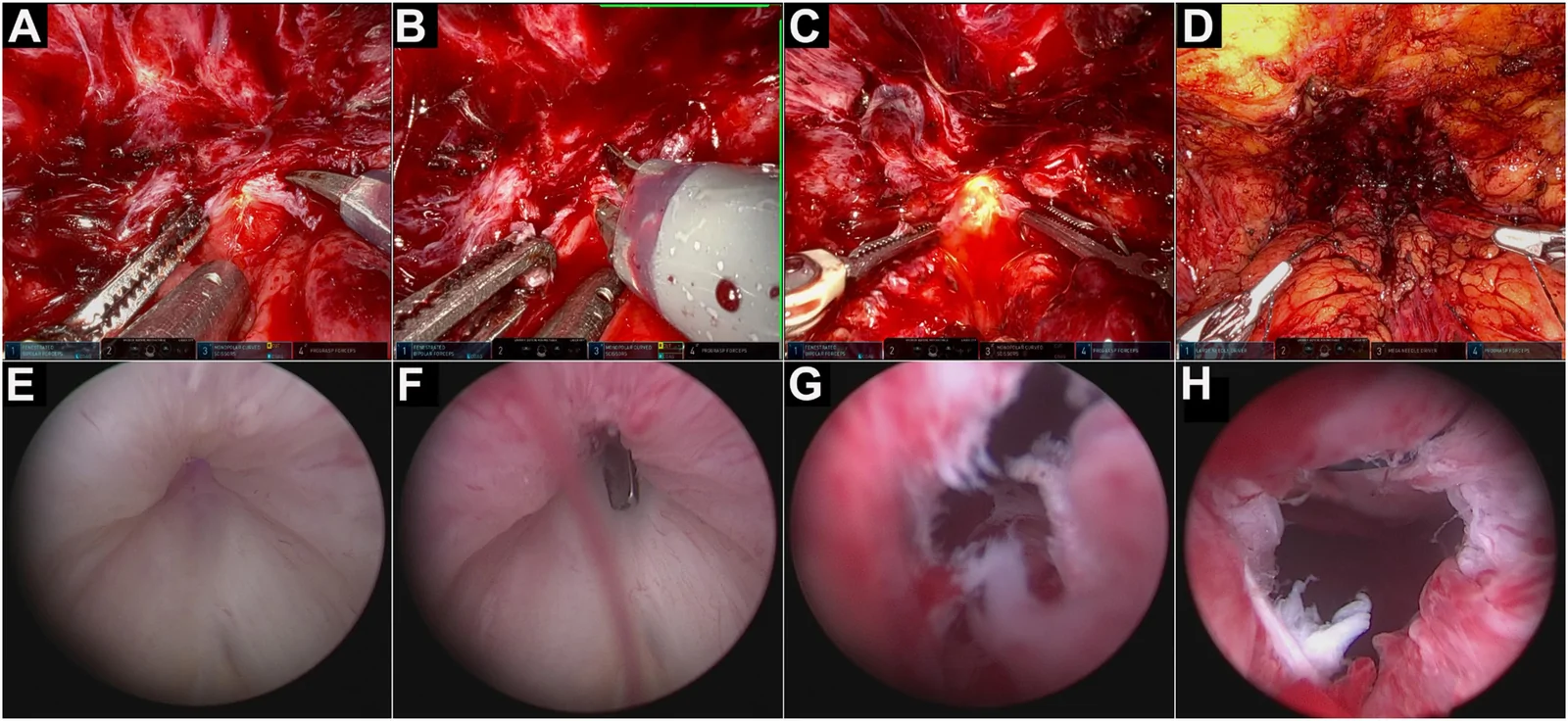
The surgical procedures.

A) The bladder is first freed from the overlying peritoneum to identify the obstructed bladder neck. B) The obstructed bladder neck was incised by monopolar scissors. C) A Y- shaped incision of the obstructed bladder neck is performed. D) A V-shaped suture is made to reconstruct na enlarged bladder neck. E) The obstructed bladder neck was identified under the guidance of urethroscopy. F) A cystoscope is placed at the membranous urethra to confirm the integrity of external urethral sphincter and observe the bladder neck incision. G: While a Y-shaped incision is performed, a cystoscope was used to observe and avoid potential injury to the external sphincter. H: The V-shaped suture is completed under cystoscopic surveillance.

**WATCH THE FULL VIDEO 1**

The apex of the V-shaped anterior bladder flap is advanced into the distal aspect of the incision, and a V-shaped suture is made to reconstruct an enlarged bladder neck using 3-0 barbed sutures (Figure-2D). The suturing of the apex is also completed under cystoscopic surveillance (Figure-2H and Supplementary video 2).

**WATCH THE FULL VIDEO 2**

After control for leak tightness, an F22 silicone catheter is inserted into the urethra to maintain patency.

### Postoperative management and follow-up

Urethral catheter was routinely left indwelling for two weeks after surgery. After catheter removal, patients were scheduled for re-examination at one, six, twelve and twenty-four months. After two years, all patients were consistently followed up through outpatient visits, telephone calls, or e-mails. Uroflowmetry was routinely performed. Cystoscopy was performed at six months after surgery (Supplementary video 3).

**WATCH THE FULL VIDEO 3**

RVCUG was not performed for everyone. However, if the patient complained of urinary obstruction during follow-up, RVCUG became necessary. Success of VUAS after cystoscopy-guided robotic Y-V plasty was defined as restoring a wide bladder neck determined by flexible cystoscopy and no need for further intervention procedures such as dilatation, internal urethrotomy, or reconstructive surgery. The surgery was considered successful if the maximum urine flow rate is greater than 15mL/s and the 17Fr cystoscope can pass smoothly. The Clavien–Dindo Classification system was employed to categorize postoperative complications (17). All patients were followed up on urinary continence, erectile function and IPSS at three and six months postoperatively in the outpatient clinic. Based on symptoms, urinary continence was categorized into three levels: normal continence, social continence defined as a requirement of 2 pads or less per 24-hour period, and clinical incontinence necessitating more than 2 pads per 24-hour period (18).

## RESULTS

### Patient characteristics

In total, 31 patients diagnosed with VUAS after radical prostatectomy had undergone cystoscopy-guided robotic Y-V plasty. Three patients were excluded because of incomplete follow-up data. Therefore, 28 patients with a median age of 69 (range 60–85) years were finally enrolled. Table-1 presents the demographics and clinical data, including age, body mass index, diabetes mellitus, previous surgical approach to radical prostatectomy, previous surgical methods to radical prostatectomy, previous pelvic lymph node dissection, history of failed transurethral procedures, suprapubic cystostomy, erectile dysfunction, disease course and VUAS length. The operation time was 50–150 (median 70) min. Intraoperative blood loss was 50–200 (median 80) mL.

**Table 1:** Patient demographics and clinical records.

Variables		Value
Patients, n		28
Age, median (range) yrs		69 (60 - 85)
BMI (kg/m2), median (range)		26.4 (19.2 - 32.5)
**Diabetes mellitus, n (%)**		
	Yes	5 (17.9)
	No	23 (82.1)
**Previous surgical approach to RP, n (%)**		
	Extraperitoneal	20 (71.4)
	Transperitoneal	8 (28.6)
**Previous surgical methods of RP, n (%)**		
	Laparoscopic	17 (60.7)
	Robotic	11 (39.3)
**Previous pelvic lymph node dissection, n (%)**		
	Yes	2 (8.1)
	No	26 (92.9)
**History of failed transurethral procedures, n (%)**		
	Urethral dilatation	19 (67.9)
	DVIU	22 (78.6)
	TUR of BN	20 (71.4)
	Any two or more procedures	28 (100)
**Suprapubic cystostomy, n (%)**		
	Yes	7 (25.0)
	No	21 (75.0)
**Erectile function**		
	Normal	0 (0)
	Mild ED	1(3.6)
	Moderate ED	2(7.1)
	Severe ED	25 (89.3)
Disease course, median (range) months		21 (7 - 47)
Stenosis length, Mean ± SD (range) mm		5.32 ± 0.98 (4 - 8)
VUAS recurrence, n (%)		1 (3.6)
**Postoperative complications, Clavien-Dindo, n (%)**		
	I	8 (28.6)
	II	3 (10.7)
	II	0
	VI	0
	V	0
	Any grade	11 (39.3)
**Erectile function, n (%)**		
	Normal	0 (0)
	Mild ED	1(3.7)
	Moderate ED	2(7.4)
	Severe ED	24 (88.9)
	Total	27 (100)
**Urinary continence, n (%)**		
	Normal continence	24 (92.6)
	Social continence	2 (7.4)
	Clinical incontinence	0 (0)
	Total	27 (100)

BMI = body mass index; BN = bladder neck; DVIU = direct visual internal urethrotomy; ED = erectile dysfunction; RP = radical prostatectomy; TUR = transurethral resection.

### Treatment effectiveness

All patients were followed up for 6–59 (median 30) months. After surgery, the final success rate was 96.4% (27/28). Postoperative complications mainly included bladder spasm in six patients, urethral orifice pain in two, wound infection in one, wound bleeding in one and intermuscular vein thrombosis of lower limbs in one. No serious complications related to cystoscopy-guided robotic Y-V plasty occurred. Normal continence was achieved in 25 patients (92.6%). Social continence occurred in 2 patients (7.4%). No patient experienced clinical incontinence. The incidence of ED was similar to that before surgery (Table-1). Among the 27 patients who were successfully treated, the maximum urinary flow rate (Qmax) was significantly improved, with preoperative and postoperative Qmax of 7.1 ± 1.7 mL/s and 18.1 ± 2.8 mL/s respectively (p=0.000). Likewise, the patients’ IPSS scores also achieved significant improvement with preoperative and postoperative scores of 19.1 ± 1.8 and 5.9 ± 1.7 respectively (p=0.000). The failed patient was successfully cured with transurethral resection of bladder neck.

## DISCUSSION

With the aging of the social population and the increasing detection rate of prostate cancer year by year, especially Asian countries (19, 20), it is urgent to find an effective surgical method to solve the VUAS after radical prostatectomy. In this work, the new surgical strategy of cystoscopy-guided robotic Y-V plasty was preliminarily effective in treating VUAS following radical prostatectomy. It not only ensures a high success rate but also reduces the occurrence of urinary incontinence. This report is the first on the application of cystoscopy-guided robotic Y-V plasty in the surgical treatment of VUAS.

For the treatment of patients with VUAS, while reconstructing a wide bladder neck, it is also necessary to consider how to ensure urinary control. Since the prostatic urethra is completely resected during radical prostatectomy, and the internal urethral sphincter is also destroyed, the patient's urinary control mainly relies on the external urethral sphincter. Therefore, the focus of reconstructive surgery for patients with VUAS is to protect the external urethral sphincter. According to previous reports (21), the traditional bladder neck Y-V plasty involves incision and reconstruction of the bladder neck under direct vision through a transabdominal or extraperitoneal approach (22). Although this surgery has a high success rate in resolving patients’ urinary difficulties, it cannot ensure whether the external sphincter is damaged during the surgery. The incontinence rates were about 18-27% (2). In this regard, based on this surgical procedure, we made technical improvements aimed at protecting the external sphincter, that is to perform bladder neck Y-V plasty under direct vision of the cystoscope. Two reconstructive urologists operated da Vinci platform and cystoscope simultaneously in a combined antegrade and retrograde manner, to avoid over-incision of the bladder neck and posterior urethra, and to avoid suturing into the external sphincter. This can effectively prevent damage to the external sphincter. In this study, the surgery was performed transperitoneally because the difficulty of establishing surgical cavities and surgical procedures through an extraperitoneal pathway is extremely harder.

In this study, the surgical success rate reached 96.4%. Among the 27 patients who were successfully treated, no patient experienced severe incontinence requiring urinal pads. Only two patients developed occasional incontinence without the need for urinal pad usage. Postoperative cystoscopy showed that the contractile function of the external urethral sphincter in the two patients was not significantly damaged. According to our follow-up results, the urinary leakage symptoms of the two patients were significantly relieved in 6 and 8 months after surgery respectively. On the third to sixth day after surgery, bladder spasm occurred in six patients. At the same time, the amount of drainage in these patients increased significantly, and the creatinine of the drainage fluid also increased. Among these, five patients showed significant relief in bladder spasm symptoms and corresponding reduction in drainage after conservative treatment. However, one case showed no significant improvement after 3 days of conservative treatment, so the catheter was replaced by a 16 Fr nephrostomy balloon catheter on the seventh day after surgery. Subsequently, the patient's bladder spasm symptoms were resolved.

Each surgical technique has its indications. In recent years, bladder neck Y-V plasty has gradually become the gold standard surgical procedure for bladder neck stenosis after prostatic hyperplasia. However, for patients with VUAS after radical prostatectomy, it is necessary to strictly select suitable patients before performing Y-V plasty. Patients whose preoperative cystoscopy indicates that the stenosis involves the external urethral sphincter are not suitable for Y-V plasty. We believe that the key to ensuring good urinary control is that there should be a certain distance between the distal end of the Y-shaped incision and the external urethral sphincter to ensure that the anastomosis will not affect the external sphincter. In patients with long stenosis zones involving the external sphincter, Y-V plasty combined with an artificial sphincter system may be a more suitable option.

A good therapeutic effect was achieved through the strategy of cystoscopy-guided robotic Y-V plasty; however, this study had some limitations. First, this is a retrospective clinical study, and there may be some bias in the collection of research data. Prospective studies are imperative for validating its effectiveness. Second, definite conclusions cannot be drawn from the small cohort. Therefore, this study only provides some references on how to reduce incontinence after bladder neck reconstruction. Future studies should aim to include a larger pool of patients to ensure greater reliability of the results. Third, the cause of social continence remains unclear. Urodynamic testing should be used to evaluate urethral pressure and bladder detrusor function as well as bladder capacity. Fourth, this study did not include patients with a history of radiotherapy, so the conclusions drawn from this study are not applicable to all VUAS patients.

We believe that the proposal of this precise surgical strategy will further improve the treatment effect of VUAS after radical prostatectomy. At the same time, we hope that researchers will jointly conduct such clinical studies to further clarify the pros and cons of this surgical strategy and improve the details of the surgical procedures.

## CONCLUSIONS

This is the first report on cystoscopy-guided robotic Y-V plasty in the surgical treatment of VUAS after radical prostatectomy. This surgical strategy is more precise, direct and efficient in protecting the external urethral sphincter. It ensures a high success rate and simultaneously reduces the occurrence of urinary incontinence. This technology is worth further validation and widespread promotion.

## Data Availability

The data presented in the study are included in the article, and further inquiries can be directed to the corresponding authors.
